# Seventh International Congress of Veterinary Surgeons at Baden-Baden, 1899

**Published:** 1898-07

**Authors:** 

**Affiliations:** President; Baden-Baden


### SEVENTH INTERNATIONAL CONGRESS OF VETERINARY SURGEONS AT BADEN-BADEN, 1899.

In accordance with the resolution of the Sixth International Congress of Veterinary Surgeons, held at Berne in 1895, the Seventh Congress will take place at Baden-Baden in the year 1899. The veterinary surgeons of Baden are intrusted with the carrying out of the arrangements. With the consent of an international meeting, held at Stuttgart in June, 1896, they have formed the undersigned Committee of Management, which has resolved to hold the Congress in Baden-Baden in the first half of August, 1899.

The programme is as follows:

Precautionary measures against the spread of epidemic diseases in consequence of international trade in animals.

The prevention of tuberculosis among domestic animals and the use of the flesh and milk of animals suffering from this disease; and, connected with this, the latest demands for an effectual meat-inspection.

The prevention of foot- and-mouth disease.

The prevention of swine fever.

The forwarding of veterinary science, especially by the erection of institutions for experiments in diseases, and by founding chairs of comparative medicine in colleges for veterinary surgeons.

Conclusion of the work of the drawing-up of a common nomenclature in veterinary medicine.

Official veterinarism.

(This programme may be altered or supplemented if generally desired.)

In the proceedings, besides the German language, English and French will be permitted. Arrangements will be made for the immediate translation of all speeches and reports.

In consideration of the great expenses connected with the Congress, the fee for members is fixed at M12=12 s. For ladies who wish to attend the Congress, ladies' tickets will be issued on application ; price, M6=6 s.

Every member, even if unable personally to be present in Baden-Baden, will receive copies of all publications of the Congress, including the General Report. The sale price of this report, which the members will receive free of charge, is fixed at M16.

Arrangements for rooms for members of the Congress will be made by a lodgings committee in Baden-Baden. We are already in a position to state that those who take part in the Congress will be able to find board and lodging from M6 per day. The town of Baden-Baden has undertaken by arrangement with the Baden Committee to provide special entertainments and festivities for the members.

The Grand-ducal Government of Baden and the Chancellor of the Empire have generously made a considerable grant toward the expenses of the Congress.

Dr. Lydtin, Geheimer Oberregierungsrath, Lichtenthalerstrasse 9 Baden-Baden, will be happy to give any further information which may be desired.

The Filiale der Rheinischen Creditbank in Baden-Baden will act as treasurer.

For information respecting lodgings apply to the Ortsausauschuss des Seventh Internat. Thierarztl. Kongresses, Lichtenthalerstrasse 9, Baden-Baden.

The committee of management, in issuing invitations to the Congress, feels it may safely assure those who desire to take part in it that the time spent in Baden-Baden will not only be of the greatest professional importance, but will also offer the participants the pleasures and amusements of a first-class watering-place.

In the name of the Committee of Management of the Seventh International Congress of Veterinary Surgeons,

DR. LYDTIN,
President.

BADEN-BADEN, 15th June, 1898.

